# Notch2 Signaling Regulates the Proliferation of Murine Bone Marrow-Derived Mesenchymal Stem/Stromal Cells via c-Myc Expression

**DOI:** 10.1371/journal.pone.0165946

**Published:** 2016-11-17

**Authors:** Yukio Sato, Yo Mabuchi, Kenichi Miyamoto, Daisuke Araki, Kunimichi Niibe, Diarmaid D. Houlihan, Satoru Morikawa, Taneaki Nakagawa, Toshihiro Nakajima, Chihiro Akazawa, Shingo Hori, Hideyuki Okano, Yumi Matsuzaki

**Affiliations:** 1 Department of Emergency and Critical Care Medicine, Keio University School of Medicine, Tokyo 160-8582, Japan; 2 Department of Physiology, Keio University School of Medicine, Tokyo 160-8582, Japan; 3 Institute of Medical Science, Tokyo Medical University, Tokyo 160-0023, Japan; 4 Tokyo Medical and Dental University, Graduate School of Health Care Sciences, Department of Biochemistry and Biophysics, Tokyo 113-8510, Japan; 5 Shimane University Faculty of Medicine, Department of Life Science, Shimane 693-8501, Japan; 6 Department of Dentistry and Oral Surgery, Keio University School of Medicine, Tokyo 160-8582, Japan; 7 Division of Molecular and Regenerative Prosthodontics, Tohoku University Graduate School of Dentistry, Miyagi 980-8575, Japan; 8 Centre for Liver Research, NIHR Biomedical Research Unit, University of Birmingham, Birmingham B15 2TT, United Kingdom; The University of Adelaide, AUSTRALIA

## Abstract

Mesenchymal stem/stromal cells (MSCs) reside in the bone marrow and maintain their stemness under hypoxic conditions. However, the mechanism underlying the effects of hypoxia on MSCs remains to be elucidated. This study attempted to uncover the signaling pathway of MSC proliferation. Under low-oxygen culture conditions, MSCs maintained their proliferation and differentiation abilities for a long term. The Notch2 receptor was up-regulated in MSCs under hypoxic conditions. Notch2-knockdown (Notch2-KD) MSCs lost their cellular proliferation ability and showed reduced gene expression of hypoxia-inducible transcription factor (HIF)-1α, HIF*-2*α, and c-Myc. Overexpression of the c-Myc gene in Notch2-KD MSCs allowed the cells to regain their proliferation capacity. These results suggested that Notch2 signaling is linked to c-Myc expression and plays a key role in the regulation of MSC proliferation. Our findings provide important knowledge for elucidating the self-replication competence of MSCs in the bone marrow microenvironment.

## Introduction

Mesenchymal stem/stromal cells (MSCs) have self-renewal and multipotent differentiation abilities *in vitro* and are an attractive candidate for regenerative medicine strategies [1]. Therefore, several clinical studies utilizing MSCs in degenerative diseases are underway all over the world. MSCs were identified in the bone marrow (BM) [2], dental pulp [3], adipose tissue [4], synovium [5], and other tissues [6] based on their ability to form colony-forming unit fibroblasts (CFU-Fs) and their surface markers [7, 8]. CFU-Fs have the potential to differentiate into osteoblasts, adipocytes, and chondrocytes [1, 9]. MSCs have been obtained from cell culture studies using normoxic conditions (an oxygen concentration of ~20%). However, the local oxygen concentration in murine BM is quite low, and several studies demonstrate that culturing BM stem cells under hypoxic conditions is more advantageous for cell proliferation [10, 11]. The mechanism underlying the effects of hypoxia on MSC proliferation and differentiation abilities remains to be elucidated.

Hypoxia regulates cell division and differentiation in stem cell populations [12]. Recent reports suggest that hypoxia regulates the quiescence of hematopoietic stem cells (HSCs) residing in the BM niche [11, 13]. Moreover, hypoxia-inducible transcription factors (HIFs) are increasingly recognized for their capacity to direct the homeostasis of other populations of stem cells without cellular senescence [12, 14]. Downregulation of either HIF-1a or HIF-2a dramatically affects MSC propagation and differentiation to adipocytes [10]. In addition, Notch signaling is also thought to play an important role in maintaining the undifferentiated status of stem cells [15–17]. Myogenic cell lines are maintained the immature state under hypoxic condition through crosstalk with Notch signaling [18]. Deletion of Notch signaling components in mesenchymal tissue reduces the number of MSCs in young mice [19]. HIF-1α and Notch signaling are closely related and induce cell proliferation [20]. Notch expression being activated through the HIF-1 under hypoxic condition [21]. Based on these results, hypoxic culture conditions and Notch signaling may be involved in maintaining MSC phenotype.

Prolonged culture of MSCs on plastic reduces their proliferative ability and causes them to change into mature phenotypes [22, 23]. MSC culture media has been supplemented with different growth factors and the culture conditions have been varied in an attempt to overcome this issue [24]. The use of hypoxic culture conditions is key methods for long-term culture of stem cells to promote proliferation and to maintain their multipotent state. In the present study, we aimed to explore whether Notch signaling is involved in the regulation of murine BM-MSC proliferation under hypoxic conditions. Using flow cytometry-based MSC isolation methods, we demonstrated that Notch2 signaling controls the proliferation of purified MSCs. Overexpression of the c-Myc gene in Notch2-knockdown (Notch2-KD) MSCs allowed the cells to regain their proliferation capacity. These data showed that Notch2-c-Myc signaling is a key factor in the regulation of MSC proliferation.

## Materials and Methods

### Animals

Adult C57BL6J wild-type mice (6–8 weeks old: female) were purchased from Sankyo Labo Service Corporation (Tokyo, Japan). All experimental procedures were approved by the Ethics Committee of Keio University (Tokyo, Japan) and were performed in accordance with the ARRIVE guidelines for reporting animal research. Animals were verified completely non-responsive to stimuli before euthanasia by cervical dislocation.

### Detection of cellular hypoxia

To evaluate the environmental oxygen conditions for MSCs in the murine BM, BM-MSCs were isolated from adult C57BL6 wild-type mice after intraperitoneal injection of pimonidazole hydrochloride (Hypoxyprobe^™^; NPI Inc., Burlington, Massachusetts, USA), a marker of hypoxia [25–27]. The compound (1.5 mg/mouse in PBS) was injected into the tail vein, and mice were sacrificed by cervical dislocation after 90 min. For flow cytometric detection, cells were stained for anti-pimonidazole fluorescein isothiocyanate (FITC)-conjugated antibodies.

### Immunofluorescence staining of BM sections

Frozen BM sections were prepared and immunostained according to the Kawamoto method, as previously reported [28]. Dissected femurs were embedded in Super Cryoembedding Medium (SCEM) and fixed using dry ice and hexane. Bones were cryosectioned (7 μm sections) using cryofilms and a CM3050s cryostat (Leica Biosystems Nussloch GmbH, Biberach, Germany). Immunofluorescence data were obtained and analyzed with a LSM710 confocal microscope (Carl Zeiss Japan, Tokyo, Japan). The markers and antibodies were as follows: Hoechst (a DNA marker used to detect cell nuclei), FITC-conjugated anti-mouse stem cell antigen 1 (Sca-1), phycoerythrin (PE)-conjugated anti-mouse cluster of differentiation (CD) 45, PE-conjugated anti-mouse TER-119, APC-conjugated anti-mouse platelet-derived growth factor receptor α (PDGFRα/CD140a) (eBioscience, San Diego, CA, USA), PE-conjugated anti-mouse Sca-1, and FITC-conjugated anti-pimonidazole hydrochloride.

### Isolation and identification of mouse MSCs

Bilateral femurs, tibias, and ilia were dissected and crushed with a pestle. Bone and BM fragments were incubated with mixing for 1 h at 37°C in 0.2% collagenase (Wako, Osaka, Japan)/low-glucose Dulbecco’s modified Eagle’s medium (DMEM; Nacalai Tesque, Kyoto, Japan) containing 10 mM Hepes (Nacalai Tesque) and 1% penicillin/streptomycin (P/S). Next, the cell suspension was collected, and bone and BM fragments were again crushed. The suspension was filtered through a 70-μm cell strainer and collected by centrifugation at 280 × g for 5 min at 4°C. The cells in the pellet were collected and soaked in 1 ml of water (Sigma Aldrich Co. LLC., Tokyo, Japan) for 5–10 s to lyse the red blood cells, after which time 1 ml of 2x phosphate-buffered saline (Nacalai Tesque) containing 4% fetal bovine serum (FBS; Biowest, Nuaillé, France) was added. The cells were resuspended in HBSS+ (Hanks balanced salt solution supplemented with 2% FBS, 10 mM Hepes, and 1% P/S), and the suspension was again filtered through the cell strainer. The filtered cells were then stained with the following reagents: PE-conjugated anti-mouse Sca-1, PE/cyanine 7 (Cy7)-conjugated anti-mouse CD45, PE/Cy7-conjugated anti-mouse TER-119 antigen, biotinized anti-mouse PDGFRα (eBioscience), and biotinized anti-mouse Sca-1 antibodies, as well as a streptavidin/allophycocyanin-crosslinked conjugate (Invitrogen^™^, Life Technologies Corporation, Carlsbad, CA, USA). Residual erythrocytes, debris, doublets, and dead cells were excluded by forward scatter, side scatter, and propidium iodide (PI) gating. Finally, PDGFRα^+^Sca-1^+^CD45^−^TER119^−^ (or PαS) cells were isolated by flow cytometry on a MoFlo XDP instrument (Beckman Coulter, Inc., Brea, CA, USA) [29, 30].

### Cell culture and colony formation assay

CFU-F analysis was performed to investigate the MSC proliferation ability as described previously [29]. Briefly, freshly isolated PαS cells were cultured for 6 weeks under normoxic conditions (20% oxygen) or hypoxic conditions (1% oxygen) in culture medium (low-glucose DMEM containing 20% FBS, 10 mM Hepes, and 1% P/S). An oxygen monitoring incubator, Prescyto MG-70M (TAITEC CORPORATION, Tokyo, Japan) was used, which kept the oxygen concentration at 1%, as monitored by a sensor. In the colony formation assay, 1000 cells were seeded at the beginning of the culture in each independent experiment and were cultured for 10 days without passaging. The number of CFU-Fs (defined as colonies containing > 50 cells) was counted every week.

### In vitro differentiation assay

After freshly isolated MSCs were cultured for 1, 2, or 4 weeks, adipogenic and osteogenic differentiation assays were performed as described previously [3, 5, 29]. Cells were plated onto 96-well plates and cultured for 7 days under normoxic conditions in differentiation medium containing the components included in the following kits: Adipogenic Maintenance SingleQuots Kit^™^ (Lonza Japan, Tokyo, Japan), Adipogenic Induction SingleQuots Kit^™^ (Lonza Japan), and Osteogenic SingleQuots Kit^™^ (Lonza Japan). The medium was changed every 3 days. Osteoblast differentiation was confirmed via alkaline phosphatase staining performed with the Histofine Assay Kit (Nichirei Biosciences Inc., Tokyo, Japan). Adipocyte differentiation was confirmed by staining with Oil Red O (Muto Pure Chemicals Co., Ltd., Tokyo, Japan). Quantitative analysis of the differentiation capacity entailed microscopic observation to determine the ratio of the number of differentiated cells to the number of nuclei in each field of view (500 cells).

### Quantitative real-time PCR analysis

Cells were directly sorted into lysis buffer RLT (QIAGEN KK, Tokyo, Japan) and frozen at −80°C. RNA extraction and DNase I treatment were performed using an RNeasy Micro Kit (QIAGEN KK) according to the manufacturer’s instructions (for samples containing < 10^5^ cells). Eluted RNA samples were reverse transcribed using an AffinityScript cDNA Synthesis Kit (Agilent Technologies, Tokyo, Japan), according to the protocol supplied by the manufacturer. Quantitative real-time PCR was performed by mixing FastStart Essential DNA Green Master (Roche, Roche Diagnostics KK, Tokyo, Japan) with 5 μl of cDNA (final reaction volume, 20 μl). The reactions were performed in a LightCycler 96 System (Roche, Roche Diagnostics KK). The cycling parameters were as follows: preincubation at 95°C for 600 s, denaturation at 95°C for 10 s, annealing at 60°C for 10 s, and extension at 72°C for 10 s. Melting was conducted at 95°C for 10 s, 65°C for 60 s, and 97°C for 1 s. Amplification proceeded for 55 cycles. The PCR primers are listed in S1 Table.

### Knockdown (KD) experiments under hypoxic conditions

Notch inhibition/KD experiments were performed in BM-MSCs under hypoxic conditions using DAPT, a gamma-secretase inhibitor (at 10, 20, 40 ng/ml) to directly inhibit Notch signaling (Sigma Aldrich Co. LLC.). The control group was treated with dimethyl sulfoxide (DMSO) vehicle (1:100; Merck KGaA, Darmstadt, Germany).

Knockdown of the Notch2 gene was performed with lentiviral vectors pLKO.1-puro expressing a short hairpin RNA (shRNA) targeting mouse Notch2 (TRCN0000340451) [31], which were purchased from MISSION shRNA Library (Sigma Aldrich Co. LLC.). MSCs (2×10^5^) were cultured on a 35 mm dish. After 1 day, lentiviruses were added to the MSCs. Twenty-four hours later, the medium containing lentiviruses was removed and added the new culture medium. The transduced cells were selected by additional culture in the presence of puromycin. The scrambled shRNA control plasmid as well as pLKO.1 shRNA were purchased from the MISSION shRNA Library. CSII-EF-myc plasmids were cloned into a lentiviral vector generated in the laboratory of Dr. Miyoshi (Riken BioResource Center, Tsukuba, Japan) and used to overexpress c-Myc in BM-MSCs.

### Statistical analysis

Data are expressed as mean ± standard error of the means (SEM; n = number of culture wells). The Tukey-Kramer test was used to compare the quantification of the efficiency and the size of colonies generated among different groups. All reported *p* values were obtained using the SPSS software package (IBM^®^ SPSS^®^ Statistics version 22.0.0.0 for Macintosh), and *p* values less than 0.05 were considered significant.

## Results

### MSCs reside in the BM under hypoxic conditions

**Fig 1:**
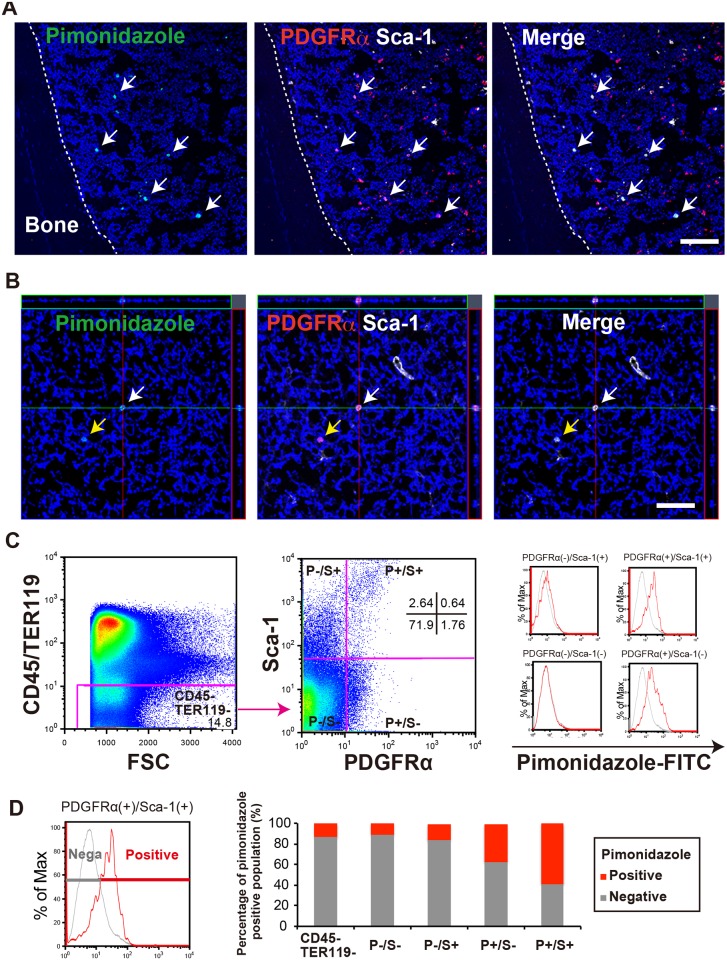
MSCs maintain a low oxidation level in the murine BM. (A) Representative IHC results for pimonidazole (green), PDGFRα (red), and Sca-1 (white), as well as Hoechst (blue) staining, in mouse BM. Scale bar = 100 μm. White arrows indicate cells co-expressing pimonidazole, PDGFRα, and Sca-1. (B) Confocal fluorescence microscopy (z-axis) images of pimonidazole-positive cells. Arrows indicate cells co-expressing the markers (white: pimonidazole high, PDGFRα, and Sca-1, yellow: pimonidazole low, PDGFRα, and Sca-1). Scale bars = 50 μm. (C) Flow cytometric analysis of pimonidazole-stained cells in the BM compartment. BM cells were split into positive-pimonidazole (Red) and negative -pimonidazole (Gray) populations, with a negative control as an index. The hematopoietic lineage negative (CD45^−^TER119^−^) and PDGFRα/Sca-1 (P^−^S^−^, P^−^S^+^, P^+^S^−^, and P^+^S^+^) populations were analyzed according to pimonidazole labeling in each fraction (n = 3). (D) Quantification of pimonidazole-stained cells in the BM compartment (n = 3).

To investigate the oxidative environment of MSCs in the BM, we performed immunohistochemistry (IHC). In our previous reports, we demonstrated that the PDGFRα^+^Sca-1^+^ (PαS) cell population is significantly enriched for murine MSCs [29, 30]. We used these markers to check the *in vivo* location and surrounding oxygen concentration of MSCs using pimonidazole. Pimonidazole is a hypoxic probe marker, and a reducing agent under hypoxic conditions (incorporated to living cells under 10 mmHg). The IHC results showed that PαS cells were stained by pimonidazole (Fig 1A and 1B). Furthermore, quantitative analysis using flow cytometry revealed that the percentage of pimonidazole-positive cells was higher in the PαS population (P^+^S^+^: higher than 60%) than in any other cell fraction in hematopoietic lineage negative (CD45^−^TER119^−^): 13%, P^−^S^−^: 11%, P^−^S^+^: 16%, P^+^S^−^: 37%) (Fig 1C and 1D). These results suggested that PDGFRα^+^Sca-1^+^ MSCs (PαS-MSCs) reside under hypoxic conditions in the BM.

### MSCs maintain proliferation and differentiation under hypoxic conditions

**Fig 2:**
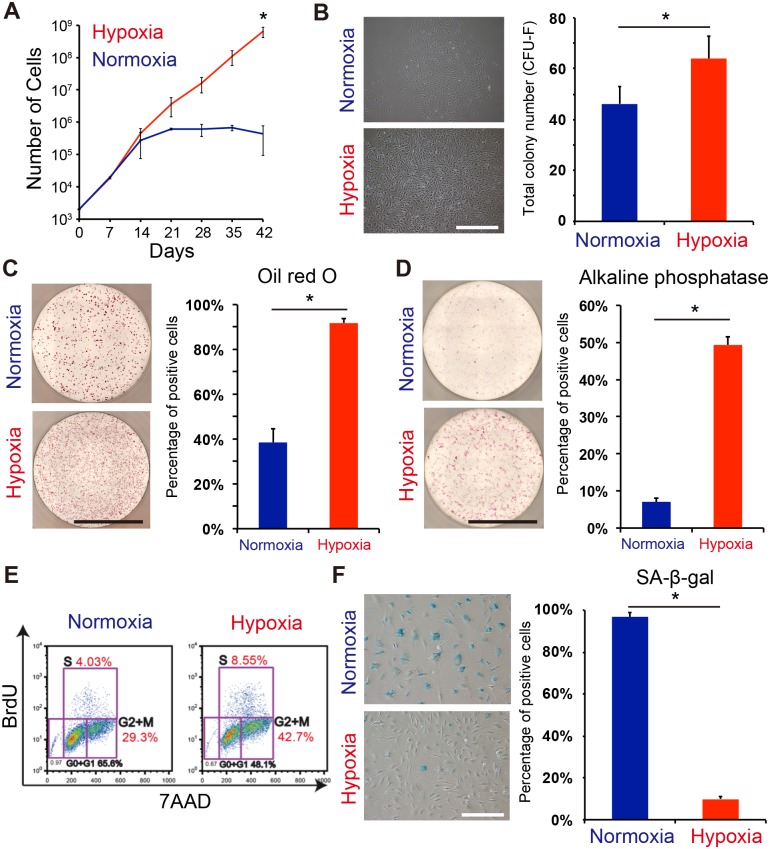
MSC potency is enhanced under hypoxic conditions. (A) Growth curve of MSCs (P^+^S^+^ cells) under normoxic (blue) and hypoxic (red) conditions. (B) Numbers of CFU-Fs detected at 14 days after plating 1000 cells (n = 3). Phase-contrast micrographs of MSC colonies are shown. The small box represents a high-resolution image (scale bar = 50 μm). (C, D) Capacity of MSCs to differentiate into adipocytes and osteoblasts (scale bar = 3.5 mm). Quantitative analysis of the MSC differentiation capacity at passage 4 (n = 3). (E) Flow cytometric analysis of BrdU/7AAD-stained P^+^S^+^ cells under normoxic (left) and hypoxic (right) conditions at passage 4. (F) SA-β-gal assay of cultured MSCs. Senescent cells were counted for quantitative analysis (n = 3, scale bar = 50 μm).

Under normal (normoxic) culture conditions, MSCs gradually lose the ability to proliferation over several passages as they become more senescent. To evaluate whether MSC stemness is maintained by hypoxia, PαS-MSCs were cultured under normoxic or hypoxic conditions, and then passaged once a week for six passages. Under normoxic conditions, the cells lost their ability to proliferate within four passages (Fig 2A, blue line). Under hypoxic conditions, the cells continued to proliferate over six passages (Fig 2A, red line). In the CFU-F assay, hypoxia-cultured PαS-MSCs generated a larger number of CFU-Fs than normoxia-cultured PαS-MSCs in primary cultures (Normoxia: 46±7 colonies, Hypoxia: 64±9 colonies) (Fig 2B). Next, we checked the differentiation capacity for adipocytes and osteoblasts in hypoxic conditions. Hypoxic PαS-MSCs maintained their capacity for both adipocytic and osteoblastic differentiation, whereas normoxic PαS-MSCs showed a low differentiation potential (Fig 2C and 2D). Furthermore, we analyzed the cell cycle under the different oxygen conditions by flow cytometry with bromodeoxyuridine (BrdU) and 7-aminoactinomycin C (7AAD) staining. The percentage of proliferating cells (i.e., cells at S or G2/M phase) was higher for the hypoxic PαS-MSCs than for the normoxic PαS-MSCs (Fig 2E). Senescence-associated β-galactosidase (SA-β-gal) staining strongly confirmed the reduced senescence in PαS-MSCs under hypoxic culture conditions (Normoxia: 97±2%, Hypoxia: 10±2%) (Fig 2F). These data suggested that MSCs maintain proliferation and differentiation capacity under hypoxic conditions with a reduction in cellular senescence.

To analyze the effect of hypoxia on MSCs in more detail, we next quantified the reactive oxygen species (ROS) expression. Under the normoxic condition, cell proliferation was increased by the addition of N-acetylcysteine (NAC), a glutathione precursor that removes excess ROS (S1A Fig). Conversely, cell proliferation was decreased by the addition of buthionine sulfoximine (BSO), which obstructs glutathione production under hypoxic conditions (S1A Fig). Using flow cytometer, ROS levels in the cell were reduced under hypoxic condition (S1B Fig). Furthermore, electron transmission microscopy showed that the number of mitochondria increased in the cell body under normoxic conditions, but not under hypoxic conditions (S2 Fig). Taken together, hypoxic conditions might promote mitophagy, preventing the generation of ROS. These data also supported that the oxygen concentration affects the maintenance of MSC proliferation.

### Notch2 signaling is required for MSC proliferation

**Fig 3:**
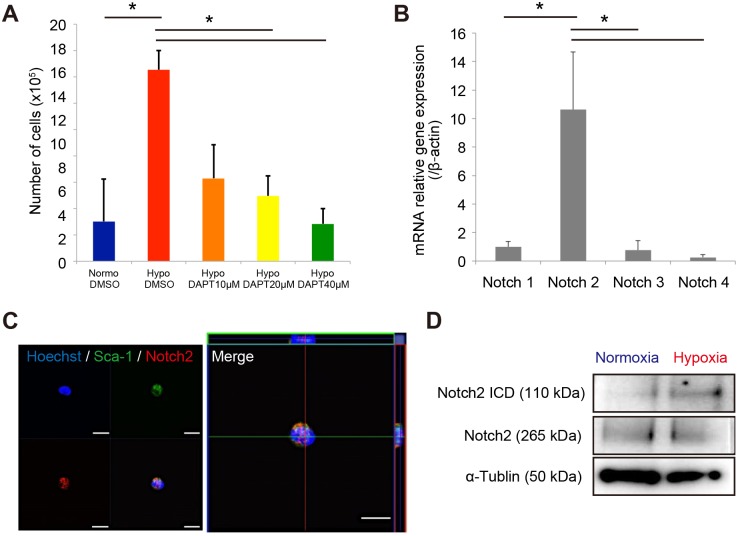
Notch signaling is upregulated in MSCs under hypoxic condition. (A) The effect of the Notch inhibitor DAPT on the MSC proliferation capacity. The normoxic group without DAPT treatment served as a control. (B) Quantitative real-time PCR of Notch family receptors (Notch1–4) in freshly sorted MSCs (P^+^S^+^ cells) (n = 3). (C) Immunocytochemistry staining of Sca-1 (green), Notch2 (red), and DAPI (blue) in freshly sorted P^+^S^+^ cells. (D) Western blotting of Notch2 ICD (ab52302, Abcam) and Notch2 full-length (D76A6, Sigma) expression in cultured P^+^S^+^ cells under hypoxic and normoxic conditions. α-tubulin (T9026, Cell signaling) served as a control. ICD, intracellular domain.

Notch signaling is crucial for stem cell maintenance [32–35]. To investigate whether Notch signaling affects the MSC proliferation ability, we analyzed the effect of DAPT (a proteolytic inhibitor of Notch) on PαS-MSCs. Inhibition of Notch signaling by DAPT treatment influenced proliferation under hypoxic conditions in a dose-dependent manner (Fig 3A). These results suggested that Notch signaling is required to induce MSC proliferation. To investigate the key signaling for MSCs, we next analyzed the expression of Notch receptors. The expression level of Notch receptor 2 was higher than those of other Notch family receptors (Notch1, 3, and 4) (Fig 3B) in freshly isolated PαS cells. The presence of PαS cells expressing Notch2 was confirmed by single cell sorting with immunocytochemical staining (Fig 3C). Notch signaling requires ligand-induce proteolytic release of intracellular domain. Cleaved Notch2 intracellular domain was also detected and increased in cultured PαS-MSCs under hypoxic condition (Fig 3D). These results suggested that Notch2 is one of the key signaling molecules for MSC proliferation.

### Notch2 signaling promotes cell proliferation via c-Myc expression

**Fig 4:**
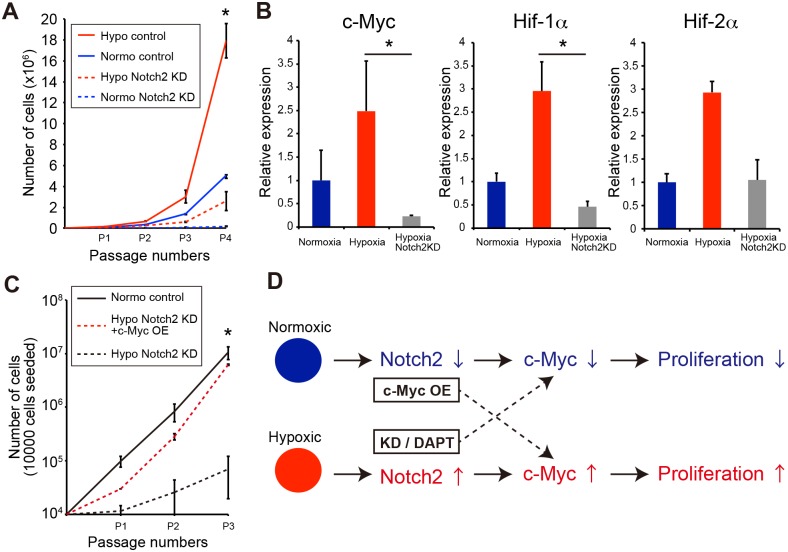
Hypoxia induces c-Myc expression via Notch2 signaling. (A) Proliferation assay of Notch2-KD MSCs. MSCs were cultured under low- or high-oxygen conditions. Notch2 KD was performed using shRNA methods. Control shRNA = scrambled shRNA. (B) Quantitative real-time PCR assay of c-Myc, HIF-1, and HIF-2 gene expression under hypoxic conditions. Normoxia served as a control. (C) Overexpression of c-Myc in Notch2-KD MSCs. Notch2-KD PαS-MSCs with or without c-Myc overexpression was cultured for three passages. (D) Schema of MSC proliferation under low- or high-oxygen conditions. Notch2 expression, together with the downstream expression of c-Myc, is higher under hypoxic conditions than under normoxic conditions. However, c-Myc expression is decreased by a Notch signaling inhibitor (DAPT), leading to the progression of senescence. Overexpression of c-Myc promotes cell proliferation and delays cell aging.

To identify the mechanism underlying MSC proliferation, we next performed a KD assay under varying oxygen culture conditions. PαS-MSC proliferation was significantly disturbed by Notch2-KD under hypoxic and normoxic conditions (Fig 4A). Moreover, the c-Myc, HIF-1α, and HIF-2α (known as interaction molecules of Notch signaling) mRNA was decreased (Fig 4B). Overexpression of c-Myc in Notch2-KD MSCs recovered the proliferation capacity *in vitro* (Fig 4C). These results suggest that the Notch2 signaling pathway regulates cell proliferation and maintenance of MSCs via c-Myc expression (Fig 4D).

## Discussion

The present study demonstrates that the Notch2 signaling pathway in BM-MSCs is essential during stem cell proliferation and maintenance. Recent reports suggest that hypoxia maintains the stemness of various types of cells, including adipose-derived stem cells [36], embryonic stem cells [37, 38], and cancer cells [39]. In the BM, HSCs maintain intracellular hypoxia and stabilize HIF-1α, where the latter is required for the preservation of HSC stemness [11, 13]. HIFs also regulate hematopoiesis in a non-cell autonomous manner by inhibiting activation of a latent program in mesenchymal progenitors that promotes hematopoiesis [10]. In our report, HIF-1α and HIF-2α mRNAs were downregulated in Notch2-KD MSCs, implying that Notch2 is located upstream of HIF. It was reported that mesenchymal progenitor proliferation *in vitro* is dependent on activation of the Notch cascade, whereas bone differentiation is enhanced by transiently suppressing this pathway [19]. These results support a model where Notch signaling in the BM maintains the stemness of a pool of MSCs by suppressing osteoblast differentiation.

Notch signaling pathways are important in MSC biology. Jagged1 (JAG1) is a Notch2 receptor ligand and most likely plays a role in activating this pathway [40]. For example, transient JAG1-mediated Notch signaling promotes the maintenance and expansion of human BM-MSCs, and concomitantly increases their skeletogenic differentiation capacity *in vitro* and *in vivo* [34]. The involvement of JAG1 in cell proliferation has also been described in breast cancer stem cells [41]. Furthermore, while Notch signaling appears to promote BM-MSCs proliferation, it also suppresses osteoblastic differentiation *in vivo* [19]. The clinical relevance of these BM-MSCs signaling pathways may be seen in two rare syndromes. Hajdu-Cheney syndrome is an autosomal dominant skeletal disorder characterized by facial anomalies, osteoporosis, and acro-osteolysis caused by truncating mutations in the last exon of Notch2 [42]. Alagille syndrome is characterized by developmental abnormalities in a variety of organs including the liver and heart [43]. The frequency of identifiable genetic mutations in patients with a clinically consistent diagnosis of Alagille syndrome is high, with JAG1 mutations identified in 94% and Notch2 mutations in 2% of affected individuals [43]. In a mouse model, Notch2 mutations disrupt osteogenesis and suppress the differentiation of functional osteoblasts [44]. Our current findings imply that Notch2-c-Myc signaling was required for proliferation by inhibiting cellular senescence under hypoxic condition *in vitro*. We also showed that MSCs (PaS cells) were resided in BM under the hypoxic condition. Although, further studies are required to examine, the pathway may prove to be a therapeutic target to treat- Hajdu-Cheney syndrome and Alagille syndrome.

Notch signaling is a key regulator of cell fate specification and cancer development. Lentivirus-mediated expression of NICD2 and c-Myc could promote proliferation of granulosa cells [45]. Other report shows that Notch2 is predominantly expressed in pancreatic intraepithelial neoplasia injuries and regulate the c-Myc signaling during tumor development [46]. Notch2 signaling strongly influenced the cell proliferation. However, overexpression of c-Myc had a limited effect on the recovery of MSC self-renewal. We speculate that the self-renewal capacity of MSCs is partially mediated by another unknown mechanism. Hypoxic condition read to remove the mitochondria and to reduce the ROS activity (S1 and S2 Figs). Our data also confirmed to link the cell proliferation (S1 Fig). The hypoxic condition might have massive effect for the MSC proliferation.

In conclusion, this study showed that c-Myc activation downstream of Notch2 is involved in the maintenance of MSC proliferation. Because c-Myc is an oncogene, modulation of Notch signaling in BM-MSCs cannot be directly exploited for regenerative medicine purposes. However, control of the Notch pathway can potentially be applied for expansion of other stem cells (e.g., HSCs, skeletal muscle stem cells, and cancer stem cells). Indeed, our culture method may provide a useful technique for stem cell expansion.

## Supporting Information

S1 Fig ROS expression in hypoxic and normoxic conditions. (A) Cell proliferation under hypoxic and normoxic conditions (blue bar: normoxic, red bar: hypoxic, orange bar: normoxic with NAC, and green bar: hypoxic with BSO). (B) ROS expression analysis by flow cytometry (1% O_2_: hypoxic, 20% O_2_: normoxic, control: negative control, and NAC^+^: normoxic with NAC). NAC; N-acetylcysteine, BSO; buthionine sulfoximine, ROS; reactive oxygen species.
(TIF)

S2 Fig Scanning electron microscope photographs of a cell under hypoxic and normoxic conditions. Electron transmission microscopy showed the number of mitochondria under normoxic (top) and hypoxic (bottom) conditions. The area enclosed by the black square is shown at a higher magnification in the image on the right side.
(TIF)

S1 Table List of gene-specific primers used for quantitative RT-PCR. (DOCX)

## References

[1] Pittenger MF, Mackay AM, Beck SC, Jaiswal RK, Douglas R, Mosca JD, et al Multilineage potential of adult human mesenchymal stem cells. Science. 1999;284(5411):143–7. . 10102814 10.1126/science.284.5411.143

[2] Mabuchi Y, Morikawa S, Harada S, Niibe K, Suzuki S, Renault-Mihara F, et al LNGFR(+)THY-1(+)VCAM-1(hi+) cells reveal functionally distinct subpopulations in mesenchymal stem cells. Stem cell reports. 2013;1(2):152–65. 10.1016/j.stemcr.2013.06.001 24052950 PMC3757748

[3] Yasui T, Mabuchi Y, Toriumi H, Ebine T, Niibe K, Houlihan DD, et al Purified Human Dental Pulp Stem Cells Promote Osteogenic Regeneration. Journal of dental research. 2016;95(2):206–14. 10.1177/0022034515610748 . 26494655

[4] Ong WK, Tan CS, Chan KL, Goesantoso GG, Chan XH, Chan E, et al Identification of specific cell-surface markers of adipose-derived stem cells from subcutaneous and visceral fat depots. Stem cell reports. 2014;2(2):171–9. 10.1016/j.stemcr.2014.01.002 24527391 PMC3923222

[5] Ogata Y, Mabuchi Y, Yoshida M, Suto EG, Suzuki N, Muneta T, et al Purified Human Synovium Mesenchymal Stem Cells as a Good Resource for Cartilage Regeneration. PloS one. 2015;10(6):e0129096 10.1371/journal.pone.0129096 26053045 PMC4459808

[6] Mabuchi Y, Houlihan DD, Akazawa C, Okano H, Matsuzaki Y. Prospective isolation of murine and human bone marrow mesenchymal stem cells based on surface markers. Stem cells international. 2013;2013:507301 10.1155/2013/507301 23766770 PMC3673454

[7] Mabuchi Y, Matsuzaki Y. Prospective isolation of resident adult human mesenchymal stem cell population from multiple organs. International journal of hematology. 2016;103(2):138–44. 10.1007/s12185-015-1921-y . 26676805

[8] Friedenstein AJ, Chailakhyan RK, Latsinik NV, Panasyuk AF, Keiliss-Borok IV. Stromal cells responsible for transferring the microenvironment of the hemopoietic tissues. Cloning in vitro and retransplantation in vivo. Transplantation. 1974;17(4):331–40. . 4150881 10.1097/00007890-197404000-00001

[9] Valtieri M, Sorrentino A. The mesenchymal stromal cell contribution to homeostasis. Journal of cellular physiology. 2008;217(2):296–300. 10.1002/jcp.21521 . 18615579

[10] Guarnerio J, Coltella N, Ala U, Tonon G, Pandolfi PP, Bernardi R. Bone marrow endosteal mesenchymal progenitors depend on HIF factors for maintenance and regulation of hematopoiesis. Stem cell reports. 2014;2(6):794–809. 10.1016/j.stemcr.2014.04.002 24936467 PMC4050345

[11] Takubo K, Goda N, Yamada W, Iriuchishima H, Ikeda E, Kubota Y, et al Regulation of the HIF-1alpha level is essential for hematopoietic stem cells. Cell stem cell. 2010;7(3):391–402. 10.1016/j.stem.2010.06.020 . 20804974

[12] Suda T, Takubo K, Semenza GL. Metabolic regulation of hematopoietic stem cells in the hypoxic niche. Cell stem cell. 2011;9(4):298–310. 10.1016/j.stem.2011.09.010 . 21982230

[13] Takubo K, Nagamatsu G, Kobayashi CI, Nakamura-Ishizu A, Kobayashi H, Ikeda E, et al Regulation of glycolysis by Pdk functions as a metabolic checkpoint for cell cycle quiescence in hematopoietic stem cells. Cell stem cell. 2013;12(1):49–61. 10.1016/j.stem.2012.10.011 . 23290136 PMC6592822

[14] Mohyeldin A, Garzon-Muvdi T, Quinones-Hinojosa A. Oxygen in stem cell biology: a critical component of the stem cell niche. Cell stem cell. 2010;7(2):150–61. 10.1016/j.stem.2010.07.007 . 20682444

[15] Morrison SJ, Perez SE, Qiao Z, Verdi JM, Hicks C, Weinmaster G, et al Transient Notch activation initiates an irreversible switch from neurogenesis to gliogenesis by neural crest stem cells. Cell. 2000;101(5):499–510. . 10850492 10.1016/s0092-8674(00)80860-0

[16] Pardo-Saganta A, Tata PR, Law BM, Saez B, Chow R, Prabhu M, et al Parent stem cells can serve as niches for their daughter cells. Nature. 2015;523(7562):597–601. 10.1038/nature14553 26147083 PMC4521991

[17] Kusumbe AP, Ramasamy SK, Itkin T, Mae MA, Langen UH, Betsholtz C, et al Age-dependent modulation of vascular niches for haematopoietic stem cells. Nature. 2016;532(7599):380–4. 10.1038/nature17638 27074508 PMC5035541

[18] Gustafsson MV, Zheng X, Pereira T, Gradin K, Jin S, Lundkvist J, et al Hypoxia requires notch signaling to maintain the undifferentiated cell state. Developmental cell. 2005;9(5):617–28. 10.1016/j.devcel.2005.09.010 . 16256737

[19] Hilton MJ, Tu X, Wu X, Bai S, Zhao H, Kobayashi T, et al Notch signaling maintains bone marrow mesenchymal progenitors by suppressing osteoblast differentiation. Nature medicine. 2008;14(3):306–14. 10.1038/nm1716 18297083 PMC2740725

[20] Min JH, Lee CH, Ji YW, Yeo A, Noh H, Song I, et al Activation of Dll4/Notch Signaling and Hypoxia-Inducible Factor-1 Alpha Facilitates Lymphangiogenesis in Lacrimal Glands in Dry Eye. PloS one. 2016;11(2):e0147846 10.1371/journal.pone.0147846 26828208 PMC4734677

[21] Ikezawa Y, Sakakibara-Konishi J, Mizugaki H, Oizumi S, Nishimura M. Inhibition of Notch and HIF enhances the antitumor effect of radiation in Notch expressing lung cancer. International journal of clinical oncology. 2016 10.1007/s10147-016-1031-8 . 27553958

[22] Kim J, Kang JW, Park JH, Choi Y, Choi KS, Park KD, et al Biological characterization of long-term cultured human mesenchymal stem cells. Archives of pharmacal research. 2009;32(1):117–26. 10.1007/s12272-009-1125-1 . 19183884

[23] Rombouts WJ, Ploemacher RE. Primary murine MSC show highly efficient homing to the bone marrow but lose homing ability following culture. Leukemia. 2003;17(1):160–70. 10.1038/sj.leu.2402763 . 12529674

[24] Ng F, Boucher S, Koh S, Sastry KS, Chase L, Lakshmipathy U, et al PDGF, TGF-beta, and FGF signaling is important for differentiation and growth of mesenchymal stem cells (MSCs): transcriptional profiling can identify markers and signaling pathways important in differentiation of MSCs into adipogenic, chondrogenic, and osteogenic lineages. Blood. 2008;112(2):295–307. 10.1182/blood-2007-07-103697 . 18332228

[25] Kennedy AS, Raleigh JA, Perez GM, Calkins DP, Thrall DE, Novotny DB, et al Proliferation and hypoxia in human squamous cell carcinoma of the cervix: first report of combined immunohistochemical assays. International journal of radiation oncology, biology, physics. 1997;37(4):897–905. . 9128967 10.1016/s0360-3016(96)00539-1

[26] Asosingh K, De Raeve H, de Ridder M, Storme GA, Willems A, Van Riet I, et al Role of the hypoxic bone marrow microenvironment in 5T2MM murine myeloma tumor progression. Haematologica. 2005;90(6):810–7. . 15951294

[27] Parmar K, Mauch P, Vergilio JA, Sackstein R, Down JD. Distribution of hematopoietic stem cells in the bone marrow according to regional hypoxia. Proceedings of the National Academy of Sciences of the United States of America. 2007;104(13):5431–6. 10.1073/pnas.0701152104 17374716 PMC1838452

[28] Yamazaki S, Ema H, Karlsson G, Yamaguchi T, Miyoshi H, Shioda S, et al Nonmyelinating Schwann cells maintain hematopoietic stem cell hibernation in the bone marrow niche. Cell. 2011;147(5):1146–58. 10.1016/j.cell.2011.09.053 . 22118468

[29] Houlihan DD, Mabuchi Y, Morikawa S, Niibe K, Araki D, Suzuki S, et al Isolation of mouse mesenchymal stem cells on the basis of expression of Sca-1 and PDGFR-alpha. Nature protocols. 2012;7(12):2103–11. Epub 2012/11/17. 10.1038/nprot.2012.125 . 23154782

[30] Morikawa S, Mabuchi Y, Kubota Y, Nagai Y, Niibe K, Hiratsu E, et al Prospective identification, isolation, and systemic transplantation of multipotent mesenchymal stem cells in murine bone marrow. The Journal of experimental medicine. 2009;206(11):2483–96. Epub 2009/10/21. 10.1084/jem.20091046 19841085 PMC2768869

[31] Nakano N, Nishiyama C, Yagita H, Hara M, Motomura Y, Kubo M, et al Notch signaling enhances FcepsilonRI-mediated cytokine production by mast cells through direct and indirect mechanisms. Journal of immunology. 2015;194(9):4535–44. 10.4049/jimmunol.1301850 . 25821223

[32] Muto J, Imai T, Ogawa D, Nishimoto Y, Okada Y, Mabuchi Y, et al RNA-binding protein Musashi1 modulates glioma cell growth through the post-transcriptional regulation of Notch and PI3 kinase/Akt signaling pathways. PloS one. 2012;7(3):e33431 10.1371/journal.pone.0033431 22428049 PMC3299785

[33] Tokunaga A, Kohyama J, Yoshida T, Nakao K, Sawamoto K, Okano H. Mapping spatio-temporal activation of Notch signaling during neurogenesis and gliogenesis in the developing mouse brain. Journal of neurochemistry. 2004;90(1):142–54. 10.1111/j.1471-4159.2004.02470.x . 15198674

[34] Dong Y, Long T, Wang C, Mirando AJ, Chen J, O'Keefe RJ, et al NOTCH-Mediated Maintenance and Expansion of Human Bone Marrow Stromal/Stem Cells: A Technology Designed for Orthopedic Regenerative Medicine. Stem cells translational medicine. 2014;3(12):1456–66. 10.5966/sctm.2014-0034 25368376 PMC4250205

[35] Zhu X, Tollkuhn J, Taylor H, Rosenfeld MG. Notch-Dependent Pituitary SOX2(+) Stem Cells Exhibit a Timed Functional Extinction in Regulation of the Postnatal Gland. Stem cell reports. 2015;5(6):1196–209. 10.1016/j.stemcr.2015.11.001 26651607 PMC4682291

[36] Choi JR, Pingguan-Murphy B, Wan Abas WA, Noor Azmi MA, Omar SZ, Chua KH, et al Impact of low oxygen tension on stemness, proliferation and differentiation potential of human adipose-derived stem cells. Biochemical and biophysical research communications. 2014;448(2):218–24. 10.1016/j.bbrc.2014.04.096 . 24785372

[37] Forristal CE, Christensen DR, Chinnery FE, Petruzzelli R, Parry KL, Sanchez-Elsner T, et al Environmental oxygen tension regulates the energy metabolism and self-renewal of human embryonic stem cells. PloS one. 2013;8(5):e62507 10.1371/journal.pone.0062507 23671606 PMC3645991

[38] Petruzzelli R, Christensen DR, Parry KL, Sanchez-Elsner T, Houghton FD. HIF-2alpha regulates NANOG expression in human embryonic stem cells following hypoxia and reoxygenation through the interaction with an Oct-Sox cis regulatory element. PloS one. 2014;9(10):e108309 10.1371/journal.pone.0108309 25271810 PMC4182711

[39] Santoyo-Ramos P, Likhatcheva M, Garcia-Zepeda EA, Castaneda-Patlan MC, Robles-Flores M. Hypoxia-inducible factors modulate the stemness and malignancy of colon cancer cells by playing opposite roles in canonical Wnt signaling. PloS one. 2014;9(11):e112580 10.1371/journal.pone.0112580 25396735 PMC4232394

[40] Sethi N, Dai X, Winter CG, Kang Y. Tumor-derived JAGGED1 promotes osteolytic bone metastasis of breast cancer by engaging notch signaling in bone cells. Cancer cell. 2011;19(2):192–205. 10.1016/j.ccr.2010.12.022 21295524 PMC3040415

[41] Yamamoto M, Taguchi Y, Ito-Kureha T, Semba K, Yamaguchi N, Inoue J. NF-kappaB non-cell-autonomously regulates cancer stem cell populations in the basal-like breast cancer subtype. Nature communications. 2013;4:2299 10.1038/ncomms3299 . 23934482

[42] Isidor B, Lindenbaum P, Pichon O, Bezieau S, Dina C, Jacquemont S, et al Truncating mutations in the last exon of NOTCH2 cause a rare skeletal disorder with osteoporosis. Nature genetics. 2011;43(4):306–8. 10.1038/ng.778 . 21378989

[43] McDaniell R, Warthen DM, Sanchez-Lara PA, Pai A, Krantz ID, Piccoli DA, et al NOTCH2 mutations cause Alagille syndrome, a heterogeneous disorder of the notch signaling pathway. American journal of human genetics. 2006;79(1):169–73. 10.1086/505332 16773578 PMC1474136

[44] Zanotti S, Smerdel-Ramoya A, Stadmeyer L, Durant D, Radtke F, Canalis E. Notch inhibits osteoblast differentiation and causes osteopenia. Endocrinology. 2008;149(8):3890–9. 10.1210/en.2008-0140 18420737 PMC2488209

[45] Zhang CP, Yang JL, Zhang J, Li L, Huang L, Ji SY, et al Notch signaling is involved in ovarian follicle development by regulating granulosa cell proliferation. Endocrinology. 2011;152(6):2437–47. 10.1210/en.2010-1182 . 21427220

[46] Mazur PK, Einwachter H, Lee M, Sipos B, Nakhai H, Rad R, et al Notch2 is required for progression of pancreatic intraepithelial neoplasia and development of pancreatic ductal adenocarcinoma. Proceedings of the National Academy of Sciences of the United States of America. 2010;107(30):13438–43. 10.1073/pnas.1002423107 20624967 PMC2922150

