# Supplementary figures and images for: Notch2 Signaling Regulates the Proliferation of Murine Bone Marrow-Derived Mesenchymal Stem/Stromal Cells via c-Myc Expression
Source: PLoS One. 2016 Nov 17;11(11):e0165946. doi: 10.1371/journal.pone.0165946 (PMC5113929; doi:10.1371/journal.pone.0165946)

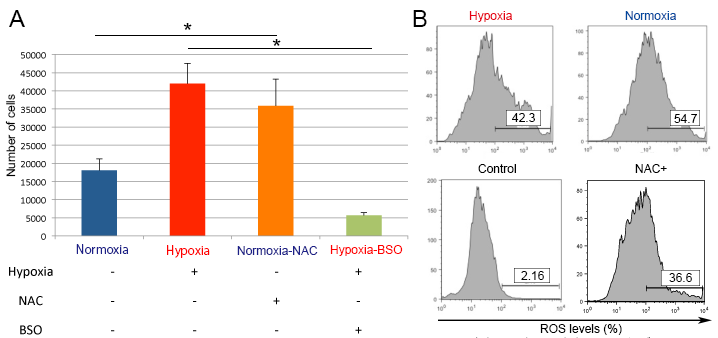

Supplement: S1 Fig — (A) Cell proliferation under hypoxic and normoxic conditions (blue bar: normoxic, red bar: hypoxic, orange bar: normoxic with NAC, and green bar: hypoxic with BSO). (B) ROS expression analysis by flow cytometry (1% O2: hypoxic, 20% O2: normoxic, control: negative control, and NAC+: normoxic with NAC). NAC; N-acetylcysteine, BSO; buthionine sulfoximine, ROS; reactive oxygen species. (TIF) [file pone.0165946.s001.tif]

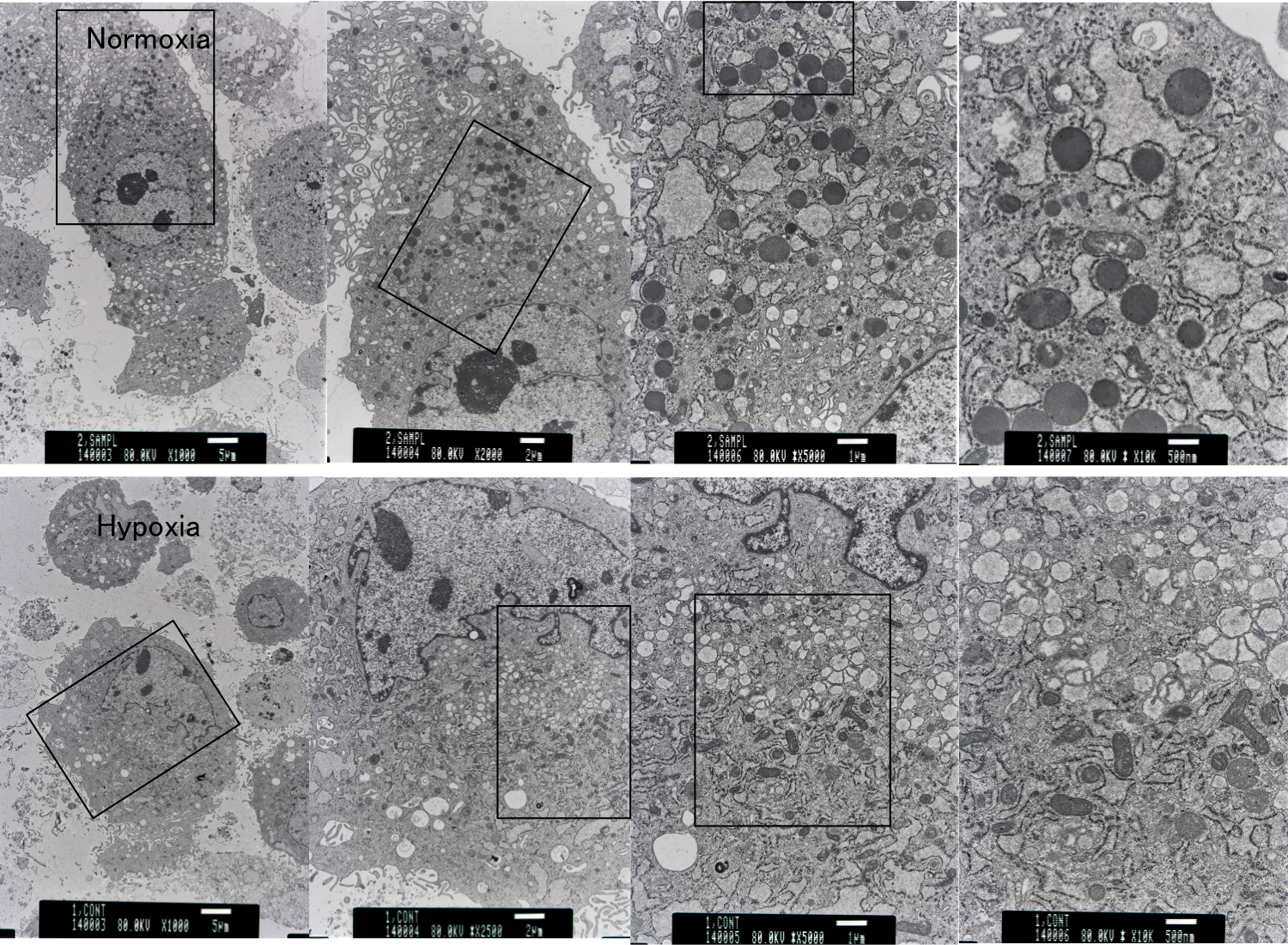

Supplement: S2 Fig — Electron transmission microscopy showed the number of mitochondria under normoxic (top) and hypoxic (bottom) conditions. The area enclosed by the black square is shown at a higher magnification in the image on the right side. (TIF) [file pone.0165946.s002.tif]
