# Supplementary material for: Notch2 Signaling Regulates the Proliferation of Murine Bone Marrow-Derived Mesenchymal Stem/Stromal Cells via c-Myc Expression
Source: PLoS One. 2016 Nov 17;11(11):e0165946. doi: 10.1371/journal.pone.0165946 (PMC5113929; doi:10.1371/journal.pone.0165946)
Supplement: S1 Table — (DOCX) [file pone.0165946.s003.docx]

**S1 Table**

| Gene  (Accession number) |  | Primer sequence |
| --- | --- | --- |
| Notch1  ([NM_008714.3](https://www.ncbi.nlm.nih.gov/nucleotide/224967064?report=genbank&log$=nucltop&blast_rank=1&RID=TMCN9BH501R)) | Sense | 5′-CGG TGA ACA ATG TGG ATG CT-3′ |
|  | Antisense | 5′-ACT TTG GCA GTC TCA TAG CT-3′ |
| Notch2  ([NM_010928.2](https://www.ncbi.nlm.nih.gov/nucleotide/134288852?report=genbank&log$=nucltop&blast_rank=1&RID=TMD5AR0301R)) | Sense | 5′-tgc aac acc gca gag tgc ct-3′ |
|  | Antisense | 5′-tcc tcg ctg ttg cat ccc tga-3′ |
| Notch3  ([NM_008716.2](https://www.ncbi.nlm.nih.gov/nucleotide/118130663?report=genbank&log$=nucltop&blast_rank=2&RID=TMD681FX01R)) | Sense | 5′-gac gga gtg gat gtc aac g-3′ |
|  | Antisense | 5′-aat gct ggc tga tgt gtc at-3′ |
| Notch4  ([NM_010929.2](https://www.ncbi.nlm.nih.gov/nucleotide/114326468?report=genbank&log$=nucltop&blast_rank=3&RID=TMD7CR1T01R)) | Sense | 5′-TGG ACC ACT GTG TGT CTG C-3′ |
|  | Antisense | 5′-GCA CAG CTG GGG TTA GTC TT-3′ |
| HIF-1α  ([NM_001313919.1](https://www.ncbi.nlm.nih.gov/nucleotide/927028880?report=genbank&log$=nucltop&blast_rank=4&RID=TMDSEDJS015)) | Sense | 5’-CCA CAG GAC AGT ACA GGA TG -3’ |
|  | Antisense | 5’-TCA AGT CGT GCT GAA TAA TAC C -3’ |
| HIF-2α (Epas1)  ([NM_010137.3](https://www.ncbi.nlm.nih.gov/nucleotide/178057355?report=genbank&log$=nucltop&blast_rank=1&RID=TMDT9G27014)) | Sense | 5’-CTA AGT GGC CTG TGG GTG AT -3’ |
|  | Antisense | 5’-GTG TCT TGG AAG GCT TGC TC-3’ |
| c-Myc  ([NM_001177352.1](https://www.ncbi.nlm.nih.gov/nucleotide/293629263?report=genbank&log$=nucltop&blast_rank=3&RID=TMDU7XSD014)) | Sense | 5’-CTG GAT TTC CTT TGG GCG TT-3’ |
|  | Antisense | 5’-TGG TGA AGT TCA CGT TGA GGG-3’ |
| β-actin  ([NM_007393.5](https://www.ncbi.nlm.nih.gov/nucleotide/930945786?report=genbank&log$=nucltop&blast_rank=1&RID=TMDVBNZW015)) | Sense | 5′-AGA CCT TCA ACA CCC CAG CCA TGT-3′ |
|  | Antisense | 5′-GGC CAG CCA GGT CCA GAC GCA G-3′ |
